# IDTWO: A Protocol for a Randomised Controlled Trial of a Web-Based Mental Health Intervention for Australians with Intellectual Disability

**DOI:** 10.3390/ijerph18052473

**Published:** 2021-03-03

**Authors:** Peter A. Baldwin, Victoria Rasmussen, Julian N. Trollor, Jenna L. Zhao, Josephine Anderson, Helen Christensen, Katherine Boydell

**Affiliations:** 1 Black Dog Institute, Sydney, NSW 2031, Australia; v.rasmussen@blackdog.org.au (V.R.); j.anderson@unsw.edu.au (J.A.); h.christensen@blackdog.org.au (H.C.); k.boydell@blackdog.org.au (K.B.); 2 Department of Developmental Disability Neuropsychiatry, School of Psychiatry, University of New South Wales, Sydney, NSW 2031, Australia; j.trollor@unsw.edu.au (J.N.T.); jenna.zhao@unsw.edu.au (J.L.Z.)

**Keywords:** intellectual disability, electronic mental health, RCT, CBT, mental health promotion

## Abstract

People with intellectual disability (ID) experience higher rates of mental illness and reduced access to appropriate care and treatment. Tailored electronic mental health (eMH) programs offer opportunities to address these disparities. The aim of this study is to examine whether a fully automated and self-guided eMH program tailored to the needs of people with ID can reduce symptoms of anxiety and depression and improve daily functioning in people with borderline-to-mild ID. Australians with borderline-to-mild ID, aged 16 years and older with mild-to-moderate depression and/or anxiety symptoms will be eligible to participate with the help of a nominated carer, if necessary. A randomised controlled trial with a sample size of 150 participants divided into treatment and waitlist control arms will be conducted. Participants randomised to the intervention group will have full access to the Healthy Mind program for eight weeks. The waitlist control group will gain full access to the program following the eight-week treatment period. Efficacy will be assessed on the Anxiety, Depression, and Mood Scale; Kessler-10; and the World Health Organisation Disability Assessment Schedule 2.0 across three time-points (baseline, eight weeks, and three months). We expect that people who use the intervention will report reduced depression and anxiety, relative to the control group. To our knowledge, this is the first study to examine the effectiveness of a fully automated eMH program for improving mental health in people with ID. We expect our study to render new knowledge on the delivery and effects of internet-based cognitive behaviour therapy (CBT) tools for people with ID.

## 1. Introduction

People with intellectual disability (ID) urgently need access to better mental healthcare [1]. Mental illness rates in this group are estimated at 2–3 times those of the general population [2,3]. Despite this prevalence, relatively few access appropriate mental health services [4]. Overlap between the cognitive and behavioural features of ID and common symptoms of mental illness can lead to diagnostic overshadowing [3,5,6,7]. Thus, symptoms of mental illness are often not properly identified, and the need for tailored or specialised mental health intervention may be underestimated.

People with ID who need mental health support face numerous barriers to accessing services [8]. ID itself poses a barrier to traditional therapies, which are designed and evaluated in neurotypical individuals [9,10], and many clinicians feel they lack the ability or training to provide appropriate care [11], which is important for effective engagement [12]. Talking to healthcare professionals can be daunting, with some individuals with ID opting to avoid physicians whenever possible [13]. People with ID are more likely to come from low-socioeconomic areas, which is both a barrier to services and a risk factor for poor mental health [3]. Australian mental health services for people with ID lag behind international best-practice guidelines [4], and they are poorly represented in mental health policy-making [14]. Though the National Disability Insurance Scheme (NDIS) offers funding for much-needed disability-related support for Australians with ID, concerns remain about the availability of appropriate mental health supports, which must be accessed through mainstream health services [15].

The impact of poor mental health in ID begins early in life. Despite experiencing higher rates of mental illness than their peers, only 1 in 10 young Australians with ID and mental illness (age rage = 5–19.5 years) receive adequate mental health care [16]. School drop-out rates for youth with ID are around one-in-three, and 50% of those who drop out cite behavioural or emotional difficulties as the primary reason [16]. Adolescence is also a time of psychiatric vulnerability [2,17] and substantial cognitive development [18,19].

Mental health has life-long implications for people with ID. Joining the workforce or living away from home for the first time are pivotal moments in early adulthood. These life transitions can be difficult for people with ID [20]. Young people with ID worry about life transitions more than their neurotypical peers, and this worry is primarily focused on coping with psychosocial distress [21]. Nevertheless, successful coping during life transitions appears to be an important factor in the long-term functioning of people with ID [22]. The ability to work and participate in their community can have wide-ranging health benefits [23]. Promoting autonomy in people with ID can help them improve problem-solving skills and feel less anxious when faced with new challenges [22]. Carers may also experience benefits to their psychological well-being [23]. The potential for wide-ranging benefits further emphasises the importance of creating accessible mental healthcare options for people with ID.

Electronic mental health (eMH) can potentially deliver tailored cognitive behaviour therapy (CBT) to thousands living with ID. This type of intervention is also uniquely placed to address many of the recommendations for adapting CBT for people with ID, such as shortened sessions, appropriately simplified language, highly visual activities, and tools that are easy for carers to use and be involved with [24,25]. eMH tools are scalable, effective, and engaging [26,27,28]. By necessity and design, online therapies are visual and interactive. The length of modules can be varied, and the time spent on each module is easily managed by the individual. Being simple and accessible, carers can easily familiarise themselves with the content and have the tool readily at hand. Effective eMH programs could also be used as an adjunct to traditional face-to-face therapy, with computer-based therapy tools improving engagement and therapeutic alliance for some clinicians and service users [13].

The present paper describes a randomised controlled trial (RCT) named the Intellectual Disability Trial for Well-being Online (IDTWO). IDTWO will evaluate the efficacy of the eMH tool Healthy Mind (Black Dog Institute, Sydney, Australia) in people with borderline-to-mild ID and mild-to-moderate symptoms of anxiety and/or depression. To our knowledge, Healthy Mind is the first self-guided and fully automated ID-specific eMH program designed in consultation with ID experts and lived-experience consultants. Healthy Mind is based on myCompass (Black Dog Institute, Sydney, Australia), an established eMH program that helps users alleviate the symptoms of depression while improving their psychosocial functioning [29]. This paper was prepared using the SPIRIT checklist for presenting clinical trial protocols and the CONSORT-EHEALTH checklist [30,31].

## 2. Objectives and Hypotheses

The primary aim of this study is to answer the research question regarding whether using a tailored eMH program (Healthy Mind) can reduce symptoms of anxiety and depression and improve daily functioning in people aged 16-years and over with borderline-to-mild ID. A secondary aim is to examine any effects that specific impairments in intellectual functioning (e.g., poor working memory) may have on engagement with, and benefits of, an eMH program. We predict that people who use the intervention will report reduced depression and anxiety, relative to the control group. We also predict that lower scores on tests of cognitive function will be related to lower engagement and benefit in the treatment group.

## 3. Trial Design

The study described here is a two-arm individually randomised controlled trial (RCT) conducted wholly online. Participants allocated to the intervention group will have full access to the Healthy Mind program for 8 weeks. Participants randomised to the control group will be placed on a waitlist for 8 weeks and given full access to the Healthy Mind program after their waitlist period expires. Participants in both groups will have uninterrupted access to treatment as usual for the duration of their study participation.

## 4. Methods

### 4.1. Participants, Interventions, and Outcomes

#### 4.1.1. Study Setting

The setting for this study is Australia. Approximately, 1–2% of the Australian population live with ID, defined as an intelligence quotient of below 70 [32,33,34,35].

#### 4.1.2. Eligibility Criteria

Participants will be eligible to participate if they are aged 16 years or over, have documented ID in the borderline or mild range, report mild-to-moderate symptoms of depression and/or anxiety, and are computer and internet literate. In addition, each study participant will be required to nominate a trusted carer to provide support throughout all trial stages. Consent, screening, and all other data collection will be completed online with carer support.

Participants will be ineligible if they have an ID diagnosis in the moderate range or above, report severe mental health problems (severe symptoms of anxiety and/or depression, severe mood disturbance and/or mania, suicidal ideation, psychotic symptoms), or are experiencing medical comorbidities or other functional impairments that might impede trial engagement (e.g., impaired vision, hearing, motor skills, etc.). Ineligible participants will be offered the opportunity to participate in the community collaboration aspect of our dissemination plan, which will involve giving lived-experience feedback on the trial results and recommendations about how future trials and services for people with ID should be designed. Any potential participant reporting severe mental health symptoms will be referred to their general practitioner for further assessment. Participants reporting acute distress will be referred to phone services offering immediate support including, Lifeline, Kids Helpline, and the Suicide Call Back Service.

#### 4.1.3. Interventions

**Figure 1:**
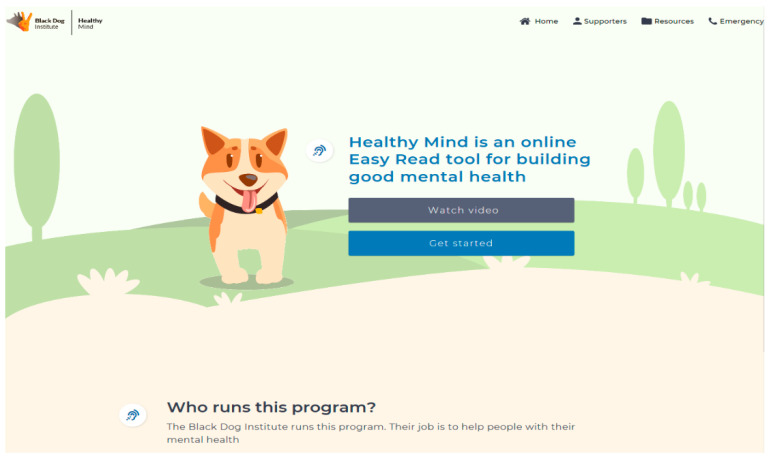
Screenshot of the Healthy Mind landing page.

**Figure 2:**
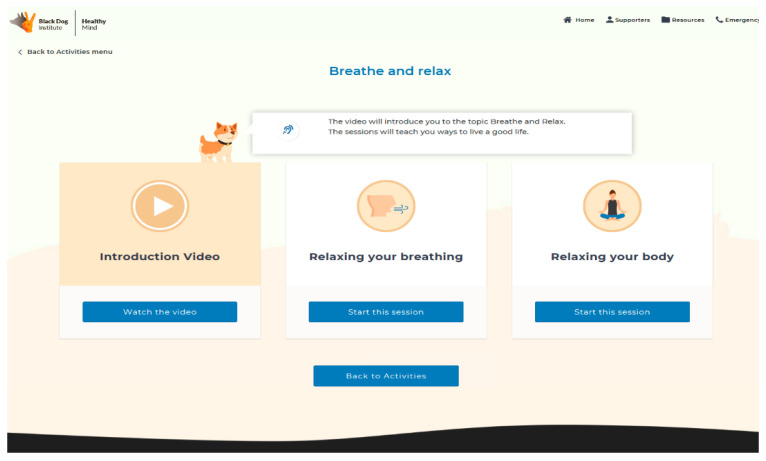
Screenshot of Healthy Mind dashboard.

Active intervention (Healthy Mind): Healthy Mind (www.healthymind.org.au) is a first-of-kind, fully automated, web-based eMH program tailored to the needs of people with ID (Figure 1). Participants randomised to the active intervention condition have access to the full Healthy Mind program for 8 weeks. Healthy Mind comprises five interactive activities (Figure 2) designed collaboratively by ID consumers and experts: (1) the “Recognising feelings” activity helps users to identify and regulate negative emotions (e.g., sad, angry, and worried); (2) “Breathe and relax” helps users understand how to use controlled breathing to calm strong emotions and be more present in their everyday life; (3) “Taming anger” offers users strategies for reducing frustration and improving communication when feeling overwhelmed; (4) “Having more fun” suggests enjoyable activities involving socialising, exercise, and leisure. The activity helps users to integrate fun activities into their regular routines, using a weekly planner; and (5) the “Tackling unhelpful thinking” activity translates core aspects of cognitive therapy into simple strategies for re-appraising stressful thoughts.

**Figure 3:**
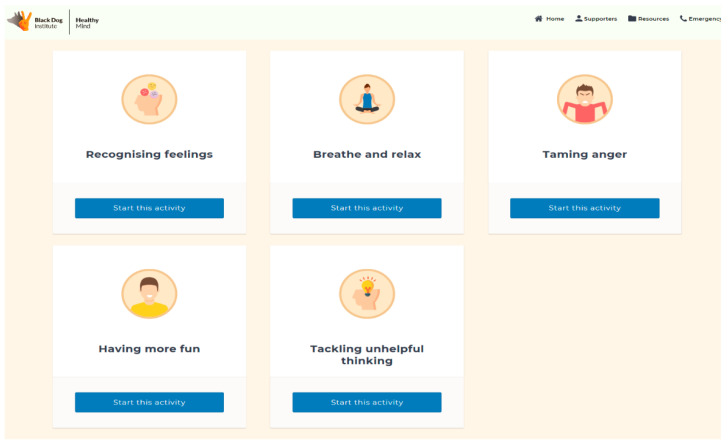
Screenshot of Healthy Mind “Breathe and relax” activity.

Each activity is presented in easy-read English and has two sessions that follow a three-step process (Figure 3): (1) “Learn about it”; (2) “Watch it”; and (3) “Do it” [36]. At step 1, users read an introduction to the topic and explanation of its impacts and value. At step 2, users watch a brief video tutorial demonstrating what the skill or behaviour looks like, enacted. At step 3, users are presented with opportunities to practise the new skill or behaviour with guided visual or audio instructions, interactive activities, and using a worksheet available for download. The program design was developed based on user feedback, elicited during the feasibility and accessibility trial [37]. For example, users requested a highly simplified structure involving early access to the videos, which were to be presented separately to the participatory components. To further enhance accessibility, users can click an icon to hear the words on the screen read aloud throughout each activity, and an animated dog is always at hand to provide support using a chat interface. Healthy Mind also offers educational and support resources for carers and emergency contact options for users who may be experiencing significant distress.

Pilot testing of Healthy Mind revealed that a sufficiently robust password system was a barrier to access due to the different language and memory abilities of users, given the site is intended for independent use by some consumers in the future [37]. Therefore, the Healthy Mind site does not require a login. User privacy is maintained by ensuring each user session is secure and unidentifiable. This is achieved by removing all opportunities to enter identifiable information (e.g., users are never asked to provide their name or any other identifiers). Identifiable information is only entered into a separate study website (detailed below).

The absence of a login system in the intervention poses a challenge for monitoring engagement with the treatment. Although this is not ideal methodologically, our priority has been to create an accessible website. This priority was identified in our co-design process previously described in our team’s publication [37]. Treatment engagement will therefore be assessed retrospectively by self-report at the first follow-up measurement.

Waitlist control group: The control group will be advised that there will be a delay in being able to access to Healthy Mind and that they will receive an email from the research team as soon as the website is available to access (i.e., after the three-month follow-up survey has closed). Control group fidelity will be maintained by collaborating with study helpers to avoid access to Health Mind and surveying control participants after the 8-week period to ensure that they were not exposed to Healthy Mind.

#### 4.1.4. Outcomes

Outcome measures will be kept to a minimum in accordance with recommendations to reduce participant demand and minimise attrition in studies involving people with ID [38]. A summary of assessments completed online by participants at baseline, immediately post-intervention, and 3-month follow-up is presented in Table 1.

Primary outcome measures: The primary outcome measure will be the Anxiety Depression and Mood Scale (ADAMS) [39]. The ADAMS is a 28-item self-report inventory that provides an assessment of psychiatric symptoms across five domains of pathology: (1) manic–hyperactive behaviour; (2) depressed mood; (3) social avoidance; (4) general anxiety; and (5) obsessive–compulsive behaviour. As both the total ADAMS score and each of the five subfactor scores demonstrate sound validity and reliability [39], efficacy of the intervention will be defined as a significant reduction in either the depressed mood or general anxiety subfactors.

Secondary outcomes: The Kessler-10 (K10) will provide an additional assessment of psychiatric symptoms [26]. The K10 is the standard measure of general psychopathology in the Australian healthcare system and thus will allow population-level comparisons between our sample and the broader Australian community.

The World Health Organisation Disability Assessment Schedule 2.0 (WHO-DAS) will provide a measure of disability and daily functioning [40]. The WHO-DAS is a 36-item self-report measure of measure health and disability across six life domains: (1) cognition; (2) mobility; (3) self-care; (4) getting along with others; (5) daily life activities, such as domestic duties; and (6) participating in society. Domain scores will be computed using the “item–response–theory” method to enable domain-level analysis across time.

Additional measurements: The autism spectrum quotient (AQ) will provide a measure of autism spectrum disorder (ASD) symptoms previously classified in the Diagnostic and Statistical Manual of Mental Disorders (DSM-IV) as characteristic of Asperger’s syndrome [41]. ASD symptoms are common in individuals with ID and may explain treatment effects [42]. The AQ is a 50-item self-report inventory that provides an overall measure of ASD symptom severity and shows excellent discriminant validity from other psychiatric disorders [41].

The CBS Health assessment system will provide an estimate of four key areas of cognitive function: (1) The Digit Span task will provide an estimate of verbal working memory; (2) the Grammatical Reasoning task will provide an estimate of the ability to use verbal information to draw conclusions; (3) the Spatial Planning task will provide a measure of the ability to use visual information to plan ahead; and (4) the Feature Match task will test the ability to focus one’s attention on specific features of an image. (https://www.cambridgebrainsciences.com/).

## 5. Participant Timeline

Participant consent, screening, and assessment will take place online via a secure study-specific website hosted on the Black Dog Institute research platform (https://healthymind.blackdoghealth.org.au), which is separate from the intervention site. The research platform is password-protected; therefore, participants may require support from their helper during this component of the study. Once on the secure site, potential participants and their helper will complete the online screening questionnaire to establish eligibility. Eligible participants and their helper will then each provide informed consent online by reviewing and agreeing to the participation information and consent statement. After providing informed consent, eligible participants will be directed to a secure website where they will enter contact details and complete the baseline questionnaires with support from their helper, then complete the CBS Health cognitive assessment independently. To allow for participant fatigue, all online assessments can be paused and resumed at a later date. Ineligible participants will receive automated feedback explaining that the study is not right for them, based on their answers. Details of telephone counselling support (e.g., Lifeline) and other self-help and face-to-face resources will be provided as appropriate.

**Figure 4:**
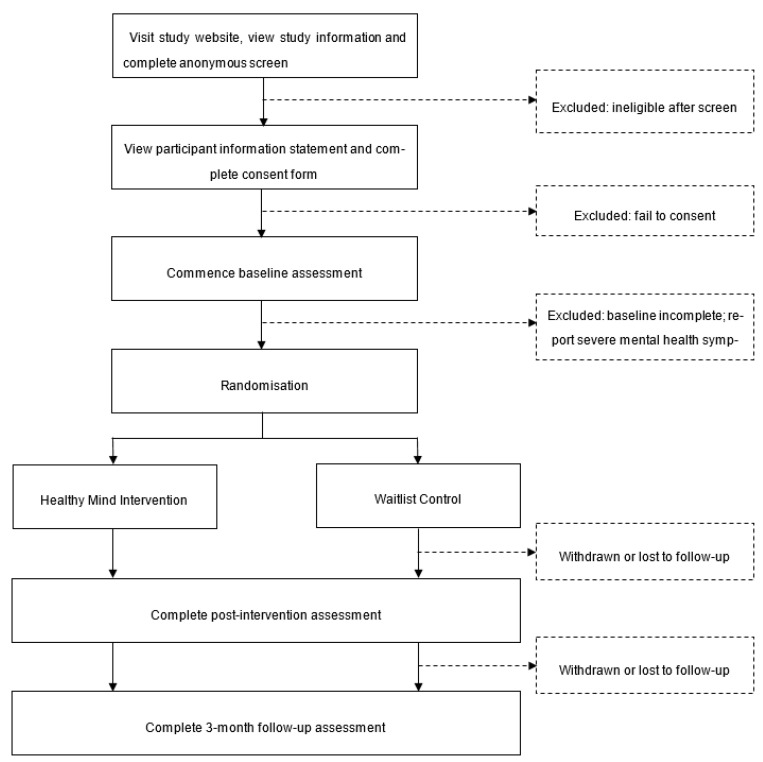
Healthy Mind CONSORT diagram.

After baseline assessment, eligible participants will be randomly allocated to one of two conditions using computerised block randomisation at a 1:1 allocation ratio: (1) the intervention condition, in which participants have full access to the Healthy Mind program; or (2) the control condition, in which participants are placed on a waitlist and given access to Healthy Mind at the conclusion of the waitlist period. Subsequent assessment points coincide for both groups at post-intervention (8 weeks) and 3 months after randomisation. The full flow of participants is presented in Figure 4.

## 6. Sample Size

The primary outcomes for this study are depression and anxiety symptoms (measured using the ADAMS). Meta-analysis of psychological therapies for people with ID suggests an expected effect of treatment on similar measures of anxiety and depression symptoms of approximately 0.6 [43]. However, this is based on face-to-face treatments and may represent an overestimation [44]. To detect a more conservative effect size of ~0.5, we will need a minimum of 50 participants per group, therefore we will recruit a sample of approximately 167 individuals to allow for ~40% attrition. This calculation was based on the group differences on ADAMS between waitlist control and intervention groups at 8 weeks and 3 months, assuming 80% power and alpha = 0.05 (0.025 for each primary group difference), in order to detect a mean difference of 0.5 standard deviation units at 8 weeks and at 3 months.

## 7. Recruitment

The study will be advertised to people aged 16-years and over with ID and their carers via the Institute’s social media channels and through promotional activities by our existing partner networks (e.g., ID researchers, clinicians, and advocacy groups). The recruitment message for the study focusses on learning new ways to manage difficult thoughts and feelings. Each study participant will also nominate a trusted carer (or carers) to provide support throughout all trial stages. Consent, screening, and all other data collection will be completed online with carer support. Diagnosis of ID will be confirmed by the nominated carer, and an estimate of cognitive functioning will be obtained using the CBS Health online cognitive assessment tool.

## 8. Assignment of Interventions

### 8.1. Allocation

Participants are allocated to a condition following a 1:1 allocation ratio using computerised blocked randomisation with blocks of eight. Randomisation occurs immediately after a participant completes the baseline assessment, ensuring allocation concealment.

### 8.2. Blinding

Participants will remain blind to study allocation during the intervention and follow-up periods; however, study helpers in the waitlist condition will be unblinded.

## 9. Data Collection, Management, and Analysis

### 9.1. Data Collection

All self-report data are being collected electronically via standardised, self-report questionnaires that are completed via the Healthy Mind study website (described above). At each assessment point, the study website automatically issues a unique link to the study questionnaire via email. Questionnaire data are kept on a secure server at University of New South Wales (UNSW), Australia, and downloaded periodically for storage in a password-protected data file accessible by P.A.B and V.R.

Cognitive function data are being collected electronically using CBS Health assessments administered via the CBS website (described above). All data will be encrypted during transmission to and storage on Amazon’s AWS EC2 service (OR, USA). Once stored, it will be accessible by only P.A.B. and V.R. on the password-protected platform. The only other individuals with access to the trial data are the CBS administrators, who may access the researcher account in case of a technical issue or emergency.

### 9.2. Retention

To maximise study engagement and retention, participants who complete questionnaires at each measurement point will receive a shopping voucher worth $20 Australian Dollars (AUD) for both themselves and their nominated carer. A scheduled reminder system is also being used to further motivate questionnaire completion. Participants and carers who do not respond to the scheduled prompt sent by email and short message service (SMS) will receive a phone call reminder from a trial team member.

### 9.3. Statistical Analysis

Statistical analyses will be performed using SPSS 25.0 (IBM Inc., Armonk, NY, USA) and MPlus (Muthen & Muthen, Los Angeles, CA, USA). Intention-to-treat analyses will be used to establish change across time and any treatment effects using linear mixed models for repeated measures (MMRM). Missing data will be handled using maximum-likelihood estimation within the MMRM procedure, as this appears to be more robust to violations of the assumption of randomness due to attrition [45]. Where appropriate, complier analyses will be used to estimate the effect of treatment-as-recommended using a complier average causal effect (CACE) procedure [46]. Cognitive sub-domain (e.g., speed of information processing) indices will be used as covariates in all analyses to determine whether cognitive profile impacts the use or efficacy of Healthy Mind. All effect sizes will be computed and reported using Cohen’s *d*.

### 9.4. Monitoring

All aspects of trial integrity, including data collection and storage, trial management, adverse events, and UNSW Human Research Ethics Committee compliance are overseen by a trial steering committee comprising authors P.A.B., K.B., J.N.T., V.R., and J.L.Z., along with members of the information technology and knowledge translation teams within the Black Dog Institute, and a lived-experience expert. The steering committee will meet bi-monthly over the lifespan of the trial. Potential adverse events may include undesired changes to mental health, which may or may not be related to the study. As the study does not impact routine care and is examining the effect of an evidence-based intervention for people with mild-to-moderate mental health symptoms (i.e., serious mental illness is an exclusion criteria), no serious adverse events are anticipated.

## 10. Ethics and Dissemination

### 10.1. Research Ethics Approval

The Healthy Mind study protocol and materials have been approved by Human Research Ethics Committee at UNSW, Australia (HC190393), and registered with the Australia and New Zealand Clinical Trials Register (ACTRN12620000113954). CI, P.A.B., will communicate with the HREC, Australian and New Zealand Clinical Trials Registry, Australian Rotary Health (ARH), and any other relevant stakeholders regarding amendments to this protocol and annual progress reports.

### 10.2. Consent or Assent

Information about the study is provided on the Healthy Mind project website. Individuals can either read the information online or download a PDF. Consent is given online by clicking a checkbox at the end of the study information page after successfully completing the eligibility screen. Participants can withdraw from the study at any time for any reason by contacting the investigation team or UNSW HREC in writing. All data collected from a withdrawn participant are securely deleted from the Black Dog Institute research platform.

### 10.3. Confidentiality

The eligibility screen is anonymous and no personal information is collected at this stage. Only eligible participants are asked to provide identifying information that is stored securely on UNSW servers and kept separate from other trial data in a password-protected file. Users are neither asked for nor able to enter identifying information into the Healthy Mind website itself.

### 10.4. Ancillary and Post-Trial Care

At the conclusion of the assigned waitlist period, the active intervention will be made available to all participants in the waitlist control group.

## 11. Discussion

Mental illness is 2–3 times more common in people with ID than in the general population, yet few access appropriate mental health services [4]. Electronic mental health (eMH) services are well placed to close this treatment gap by adapting cognitive behaviour therapy for people with ID, but very little is known about digital mental health care in ID.

The outcomes of this study will shed new light on how to support the mental health and psychosocial functioning of people with ID. Ours may be the first study to demonstrate the efficacy of a fully automated and self-guided eMH tool in people with ID, similar to those that benefit tens of thousands of other Australians [29]. Importantly, it may be the first time people with ID have a tool to effectively manage mood and anxiety independently [6]. Independence was an important theme in our user consultation [37] and supporting autonomy confers a range of mental health benefits to people with ID [22].

We expect our study will have practical implications for both treatment and policy. Positive results will provide confidence for ID clinicians to prescribe Healthy Mind as a digital treatment adjunct or psychoeducation tool, which is currently an unmet need in ID-specific face-to-face mental health services [13]. Results will also come as the Australian Royal Commission into Violence, Abuse, Neglect, and Exploitation of People with Disability (DRC) concludes and the Australian federal government formulates its National Digital Mental Health Framework. The DRC recently made its a finding that “people with cognitive disability have been and continue to be subject to systemic neglect in the Australian health system” [47]. Our trial will provide a clear example of how we might address this neglect with a cost-effective, scalable intervention like others widely available to people with more typical abilities [29].

## 12. Limitations

Volunteer bias is a possible weakness of our study, as participants who sign up may be especially motivated to learn mental health skills and engage with an eMH program designed specifically for people with ID. Problems may occur, for example, if participation is driven by a broader commitment to improving mental health, as this is likely to enhance treatment effects. However, as Healthy Mind is intended to be freely available to all Australians in the future, recruitment of motivated individuals is likely consistent with naturalistic uptake of self-help mental health programs.

Clinical trials that include people with ID often report elevated rates of attrition where participant demand is high [38], and eMH trials also experience increased study drop-out and/or disengagement from their programs [48]. Study attrition may introduce selection effects if the remaining participants share characteristics, such as especially low or high baseline symptoms scores. Our trial protocol is relatively simple, with minimal questionnaires and measures to reduce participant burden. To reduce the impact of attrition, we will recruit a larger sample than is required on the basis of our sample-size calculation and conduct between-group analyses at baseline prior to further data analysis to confirm the preservation of randomisation. If randomisation fails, variables that demonstrate systematic differences between groups at baseline will be held constant in future analyses, where appropriate. Additionally, all trial participants will have a nominated carer assist them throughout the trial, which should improve program engagement and trial completion. To further maximise trial engagement, automated program reminders will be sent by email to carers who have partially completed or failed to complete a questionnaire.

## 13. Conclusions

Access to mental health support remains a substantial challenge for many people with ID. Australian mental health services for people with ID currently fall short of international guidelines for best practice. An eMH service could play an important role in increasing access to services for this group. In establishing the efficacy of an eMH program such as Healthy Mind, we will be taking important steps towards reducing unmet treatment needs and making the mental health system more inclusive of people with ID.

  Trial registration: ACTRN126200001139

## Figures and Tables

**Table 1:** Outcome measures completed at each measurement point.

Outcomes	Baseline	Post-Intervention (8 Weeks Post Randomisation)	Follow-Up (3 Months Post Treatment Completion)
**Primary outcome measure**			
Anxiety Depression and Mood Scale (ADAMS)	X	X	X
**Secondary measures**			
Kessler 10 (K10)	X	X	X
WHO Disability Assessment Schedule (WHO-DAS)	X	X	X
Autism Quotient (AQ)	X	-	-
CBS Health Cognitive Assessment	X	-	-

## Data Availability

No new data were created or analyzed in this study. Data sharing is not applicable to this article.
